# A TOOL TO SUPPORT FAMILY AND PAID CAREGIVERS PROVIDE DAILY ORAL CARE TO PERSONS LIVING WITH DEMENTIA

**DOI:** 10.1093/geroni/igad104.1325

**Published:** 2023-12-21

**Authors:** Sato Ashida, Tim Beachy, Maria Donohoe, Haley Schneider, Hianca Pinho, Haley Wilson, Leonardo Marchini

**Affiliations:** University of Iowa College of Public Health, Iowa City, Iowa, United States; University of Iowa, Iowa City, Iowa, United States; University of Iowa College of Public Health, Iowa City, Iowa, United States; University of Iowa, Iowa City, Iowa, United States; University of Iowa College of Public Health, Iowa City, Iowa, United States; University of Iowa College of Public Health, Iowa City, Iowa, United States; Case Western Reserve University, Cleveland, Ohio, United States

## Abstract

Persons living with dementia often receive daily oral care from family caregivers or paid caregivers such as nurse aides. However, these caregivers report limited knowledge about and confidence in providing daily oral care. This study used stakeholder engaged processes to develop an app that provides information and guidance to caregivers to support their oral care provision. Contents to support oral care for persons living with dementia were developed through a collaboration between the College of Dentistry and College of Public Health based on prior research. In-depth interviews were conducted with key stakeholders including dental professionals, family caregivers, nursing home administrators, and nurse aides. Participants identified many benefits of this support tool to improve their oral care provision. Dental professionals confirmed the accuracy and importance of the information and provided additional suggestions. Family caregivers appreciated the detailed information and guidance on how to provide daily care, how to identify oral health problems, and what to do if they identify problems. Photos especially helped caregivers identify the nature of the problem to determine action steps. For paid caregivers, the app served as a good reminder and reference guide. Nursing home administrators wanted to use the app as a training tool to standardize and improve the overall care. The app was well-accepted and provided an easily accessible tool for caregivers during care provision. Additional qualitative data and pilot test results will be presented.

